# The neurotoxicity of iodoacetic acid, a byproduct of drinking water disinfection

**DOI:** 10.3389/ftox.2025.1543374

**Published:** 2025-01-27

**Authors:** Xu Wang, Chunshu Rong, Ping Niu, Wei Leng, Gaihua Wang, Ziqiao He, Xin Qi, Dexi Zhao, Jinhua Li

**Affiliations:** ^1^ Department of Encephalopathy, Hospital of Changchun University of Chinese Medicine, Changchun, Jilin, China; ^2^ School of Public Health, Jilin University, Changchun, Jilin, China; ^3^ College of Traditional Chinese Medicine, Changchun University of Chinese Medicine, Changchun, Jilin, China

**Keywords:** neurotoxicity, neurodegeneration, disinfection byproducts, iodoacetic acid, neuroinflammation

## Abstract

IAA is a by-product of the water disinfection process and has been found to be neurotoxic. However, the role and mechanism of IAA neurotoxicity remain unclear. In this review, we comprehensively discuss the neurotoxic effects and mechanisms of IAA from the molecular level, cellular level and neurological manifestations. At the molecular level, IAA causes neurotoxicity by reducing mitochondrial membrane potential, aggravating oxidative stress and DNA damage. At the cellular level, IAA causes neurotoxicity by inducing BBB disruption, neuroinflammation, and apoptosis. In neurological manifestations, IAA can lead to neurotransmitter disorders, neurodevelopment dysfunction, and even neurodegenerative diseases. Taken together, our review provides insights into the mechanisms of IAA neurotoxicity that will contribute to future studies of IAA neurotoxicity and its protective strategies.

## Graphical Abstract

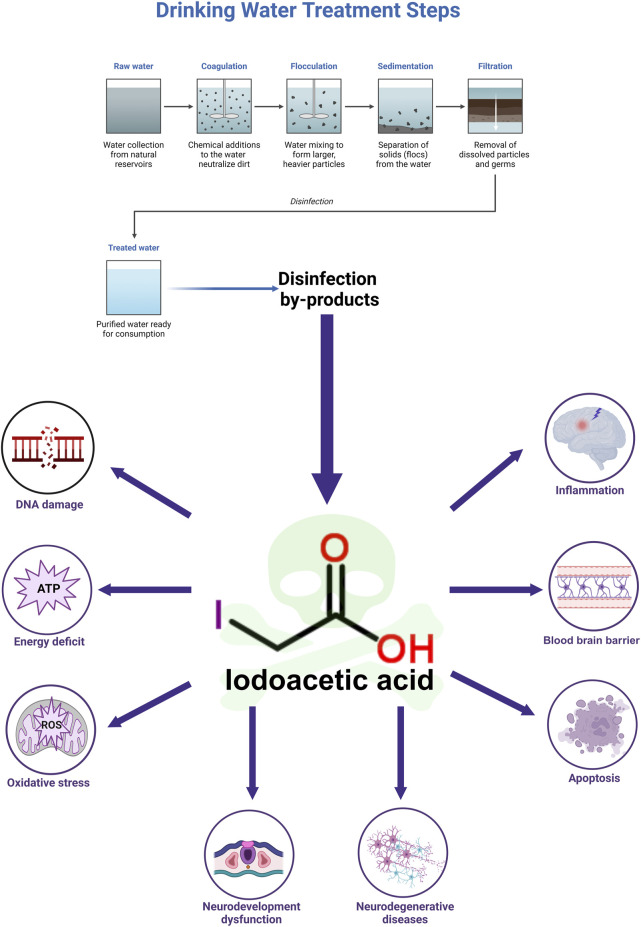

## 1 Introduction

Disinfection plays a crucial role in safeguarding public health, serving as a method that effectively eliminates bacteria, viruses, and various pathogenic microorganisms from water, thus helping to prevent the transmission of waterborne illnesses. However, this essential process can result in the creation of disinfection by-products (DBPs), one of which is iodoacetic acid (IAA). IAA is a by-product that forms as a result of the reaction between disinfectants and organic materials, as well as iodine ions present in water (MacKeown et al., 2022). A significant compound produced during this oxidation reaction is hypoiodous acid, which interacts vigorously with organic substances in the environment, ultimately resulting in the synthesis of IAA. Moreover, when chlorinated tap water interacts with salt that has been fortified with potassium iodide, the production of IAA may occur (Becalski et al., 2006). Additionally, conditions such as saltwater intrusion (Dong et al., 2019), hydraulic fracturing (Harkness et al., 2015), and the release of X-ray contrast agents contribute to the occurrence of IAA in drinking water across the globe (Li et al., 2020).

The maximum concentration of IAA identified in water samples from 23 cities across North America reached 1.7 μg/L (Richardson et al., 2008). The globally highest concentration of IAA in drinking water has yet to be established. At present, the most significant level of IAA reported in drinking water was found to be 2.18 μg/L in Shanghai, China (Wei et al., 2013). Compared to other DBPs, IAA exhibits increased cytotoxicity and genotoxicity (Wagner and Plewa, 2017). In addition, IAA disrupts intestinal flora and metabolites to exert toxicity (Sha et al., 2022). While a reference limit for IAA in drinking water has been established in China at 0.02 mg/L (Shi, 2023), it is still not regulated in either the USA or Europe (Pérez-Albaladejo et al., 2023; Yu et al., 2024). Plasma and urinary levels of IAA were measured at 1.89 ± 0.29 and 5.92 ± 1.45 ng/mL, respectively, following an oral intake of 6 mg/kg of IAA after 6 h (Yu et al., 2024). Nevertheless, there have been no investigations assessing IAA concentrations in brain tissue.

IAA is capable of traversing the blood-brain barrier (BBB), leading to neurotoxic effects (Yang et al., 2021). The phenomena of IAA mutagenesis, carcinogenesis, and teratogenesis have been thoroughly investigated (Gonsioroski et al., 2020; Zeng et al., 2021). Nonetheless, research focusing on the neurotoxic impacts and underlying mechanisms of IAA remains limited, and a comprehensive review addressing its neurotoxicity is absent. This paper represents the first review of IAA’s neurotoxicity and its associated mechanisms, establishing a groundwork for future studies in this domain.

## 2 Neurotoxicity of IAA

IAA, which inhibits glyceraldehyde-3-phosphate dehydrogenase (GAPDH), may decrease ATP levels within cells (Zhou et al., 2015). The compound can penetrate brain tissue by crossing the BBB and can lead to the destruction of nerve cells (Yang et al., 2021). IAA reduces viability of nerve cells. IAA is neurotoxic and can reduce cell viability, increase the release of lactate dehydrogenase (LDH), and even cause cell death in nerve cells (Yang et al., 2021; Chen et al., 2015; Sperling et al., 2003; Chen et al., 2017a; Lapchak et al., 2007). Compared to other tissues, brain tissue demands a higher supply of oxygen and energy (Wang et al., 2022). The mechanism by which IAA causes neurotoxicity is described below. Details of included studies and results of IAA shown in Table 1.

**TABLE 1:** Summarizing neurotoxicity of IAA.

Species	Dose	Time	Target organ	References
HT22 cells	20 μM	2 h	ROS↑, ATP↓	Suna et al. (2009)
HT22 cells	1–30 μM	2 days	Cell viability↓, Neuronal death↑, LDH↑, P2X44R↑, p-p38/p38↑, Apoptosis↑	Yang et al. (2021)
PC12 cells	50 μM	2 h	Cell viability↓, LDH↑, ROS↑, RNS↑, Apoptosis↑	Chen et al. (2015)
PC12 cells	100–400 μM	2.5 h	ROS↑,SOD↓,LDH↑,ATP↓	Sperling et al. (2003)
PC12 cells	30 μM	2 h	ROS↑,DNA damage↑,LDH↑,Bcl-2/Bax↓	Dad et al. (2013)
RGC-5 cell	2–10 μM	24 h	ROS↑, p90RSK↑, Apoptosis↑,MMP↓,caspase-3↑,caspase-7↑	Ondricek et al. (2012)
RGC-5 cells	30 μM	2 h	ROS↑, Apoptosis↑, Caspase 3↑, Cleaved PARP1↑, DNA damage↑, ATP↑, GSH↑	Maher and Hanneken (2008)
Cerebellar Purkinje cells	10–100 μM	2 h	Dopamine↓	LoPachin et al. (2006)
Rat	2.5 mM	NA	ATP↓	Sabri (1994)
HT22 cell	5 µM	2 h	Cell viability↓, ATP↓, BNDF↓, TrkB↓, p-AKT↓	Cho et al. (2021)
HT22 cell	15–30 µM	2 h	ATP↓, Oxidative stress↑, p-ERK↓, JNK↑, p38↑, p-mTOR↓, p-p70↓, p-GSK3β↓	Mata and Goldberg (2023)
HT22 cell	2.5–10 µM	2 h	ATP↓, GSH↓, Apoptosis↑	Lapchak et al. (2011)
Hippocampal cells	100 μM	2.5 h	Src↓, Fyn↓, Yes↓, LDH↓, Apoptosis↑	Shani et al. (2009)
HT22 cells	20 μM	2 h	LDH↑, Cell death↑	Lapchak et al. (2007)
Nerve cells	50 μM	4 h	ROS↑, LDH↑, Cell death↑, Apoptosis↑, Caspase 3↑, MMP↓	Zhou et al. (2015)
Nerve cells	100 μM	2.5 h	ATP↓	Rogel et al. (2005)
Astrocyte	10 μM	12 h	LDH↑, p-AKT↓, Caspase3/7↑	Gatson and Singh (2007)
Nerve cells	100 µM	2.5 h	LDH↑, IκBα↓	Kratsovnik et al. (2005)
Astrocyte	25 μM	6 h	Cell viability↓, Gap19↓, Gap26↓, Cx43↓	Kim et al. (2023)
Astrocyte	1 mM	0.5 h	Na^+^↑, Glutamic acid↓	Gemba et al. (1994)

Note: ↑ = increased, ↓ = decreased. Reactive oxygen species (ROS), lactate dehydrogenase (LDH), mitochondrial membrane potential (MMP), SOD (superoxide dismutase), reactive nitrogen species (RNS), glutathione (GSH), adenosine triphosphate (ATP).

### 2.1 Molecular level

#### 2.1.1 IAA causes energy metabolism dysfunction of nerve cells

GAPDH catalyzes the fifth step in glycolysis (Butera et al., 2018), the conversion of glyceraldehyde 3-phosphate into 1,3-biphosphoglycerate, a reaction that generates NADH, which is a “high energy” compound that subsequently generates ATP. GAPDH is best known for this reaction and is often used as a “housekeeping” gene or loading control in expression analyses, belying its complex role in growth and survival. GAPDH contains two domains for NAD^+^ binding and the catalytic domain. At the junction, cysteine 149 is a site for post-translational modification and is required for multiple GAPDH functions. While GAPDH is generally localized to the cytoplasm, it’s sub-cellular localization can change, particularly when modified by nitrosylation, oxidation and phosphorylation (Sirover, 2014).

**FIGURE 1:**
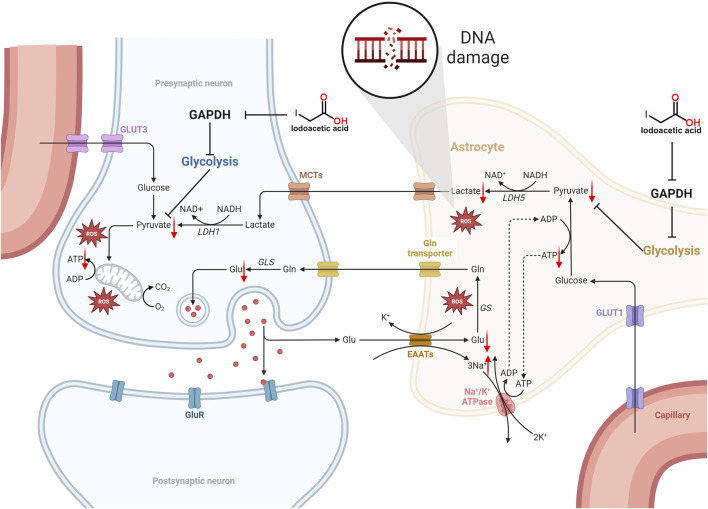
Schematic illustration of the molecular level neurotoxicity by IAA. IAA inhibited glycolysis by inhibiting GAPDH expression. The level of pyruvate is reduced, not enough to provide sufficient substrate for the tricarboxylic acid (TCA) cycle, resulting in an insufficient supply of ATP to nerve cells. In addition, ATP deficiency reduces the activity of the sodium-potassium pump, resulting in the accumulation of intracellular sodium and the decrease of extracellular potassium, which decreases the cell membrane potential. The decrease in mitochondrial membrane potential means that the electrochemical gradient between the inner and outer membrane of the mitochondria is reduced, which in turn affects ATP synthesis, and further leads to insufficient cell energy supply. In addition, lactate produced by glial cells can provide energy metabolism for neurons. Glycolysis is inhibited and lactate production is reduced. The imbalance of REDOX balance leads to the release and accumulation of excess ROS. Eventually it leads to DNA damage.

GAPDH serves as an important enzyme within the glycolytic pathway, thereby inhibiting its function will hinder the glycolytic process, which in turn impacts the synthesis of ATP (Zhu et al., 2021). The glycolytic pathway produces pyruvate, which subsequently enters the mitochondria to take part in the citric acid cycle, generating substantial ATP. Inhibition of GAPDH impacts the ensuing metabolic pathways, leading to decreased ATP synthesis. Additionally, IAA entry into the brain hampers glycolysis, consequently lowering ATP levels in nerve cells (Sperling et al., 2003; Suna et al., 2009; Maher and Hanneken, 2008; Sabri, 1994; Rogel et al., 2005). Mitochondria function as the cell’s energy production sites, generating ATP via oxidative phosphorylation. Nerve cells have very high energy requirements, and any decrease in ATP synthesis may result in compromised function and potentially lead to cell death. Moreover, a lack of ATP diminishes the sodium-potassium pump’s activity, leading to the buildup of sodium inside the cell and a decline in potassium levels outside the cell, which reduces the membrane potential. Conversely, a reduction in the potential of the mitochondrial membrane indicates a lowered electrochemical gradient across the inner and outer mitochondrial membranes, subsequently impacting ATP production and causing an inadequate energy supply for the cell (Liang et al., 2007). The specific mechanism of IAA caused molecular level neurotoxicity is shown in Figure 1.

#### 2.1.2 IAA causes oxidative stress

Oxidative stress arises from an imbalanced redox state, characterized by an excess of ROS or malfunctioning of the antioxidant systems (Ping et al., 2024). The brain is particularly sensitive to ROS due to its high requirement for oxygen and the prevalence of lipid cells that are prone to peroxide damage. Research has indicated that oxidative stress is a significant contributor to the shared pathology seen in neurodegenerative disorders. One notable aspect of oxidative stress is the heightened production of ROS, which correlates with mitochondrial dysfunction (Marchetti et al., 2006). Exposure to IAA has been shown to elevate oxidative stress levels within nerve cells. Specifically, IAA led to increased concentrations of ROS and reactive nitrogen species (RNS), along with a reduction in GSH and SOD levels in these cells (Zhou et al., 2015; Chen et al., 2015; Sperling et al., 2003; Suna et al., 2009; Maher and Hanneken, 2008). Also, IAA induces oxidative stress in germ cells via the GRP78/IRE1/XBP1s and cGAS/STING/NF-kappa B signaling pathways (Mou et al., 2024). Nonetheless, the specific molecular pathways through which IAA triggers oxidative stress in nerve cells are still not fully understood. The oxidative stress agents produced by IAA have the potential to inflict damage on DNA, resulting in compromised cellular function and potentially leading to cell death (Marchetti et al., 2006).

#### 2.1.3 IAA causes DNA damage

IAA was found to increase intracellular ROS and gamma-H2AX levels in a dose-dependent manner (Jiao et al., 2021). Heightened oxidative harm to DNA detected in both nuclear and mitochondrial DNA (mtDNA) obtained from the *postmortem* brain areas of individuals suffering from neurodegenerative disorders indicates cytogenetic injury associated with these diseases (Coppedè and Migliore, 2015). The DNA damage resulting from oxidative stress induced by IAA was observed. In retinal ganglion cells RGC-5 exposed to IAA, there was an elevation in the levels of cleaved Poly(ADP-ribose) polymerase 1 (PARP1) and p90 ribosomal s6 kinase (RSK) protein, which exacerbated the DNA damage (Maher and Hanneken, 2008). PARP1 and the PAR play a significant role in the repair of DNA damage, and a rise in the expression of cleaved PARP1 suggests a higher level of DNA damage (Ping et al., 2024). IAA additionally contributes to significant DNA damage as an inhibitor of GAPDH. Furthermore, GAPDH interacts with the DNA repair protein PARP1 (Nakajima et al., 2015). PARP1 plays a crucial role in repairing single strand DNA breaks as well as double strand DNA breaks (Ping et al., 2024). PARP1 also has the ability to ADP-ribosylate and deactivate GAPDH, which results in a reduction of GAPDH levels and may, in certain situations, lead to cell death (Du et al., 2003). Thus, IAA causes DNA damage by increasing PARP1 activity (Maher and Hanneken, 2008). The buildup of DNA damage and the malfunctioning of DNA repair mechanisms play a role in the development of various neurodegenerative disorders. Pathways that respond to DNA damage create the necessary environment to initiate cellular senescence and/or apoptosis when the extent of DNA damage is excessive. Although double-strand DNA breaks are infrequent occurrences, they represent the most lethal type of DNA damage. In neuronal cells, these double-strand DNA breaks prove especially harmful due to their diminished ability to repair DNA in comparison to proliferating cells (Merlo et al., 2016). Therefore, DNA damage is closely associated with neurodegeneration.

mtDNA, the genetic material found within this cellular energy factory, is an essential component of cellular life processes (Vazquez-Villasenor et al., 2021; Christie and Beekman, 2017). Mitochondria play a crucial role in energy conversion, and the integrity of their function is essential for maintaining normal cellular operations and energy metabolism. IAA-induced oxidative stress triggers excessive activation of mitochondrial complex I in response to persistent DNA damage. These altered responses to continuous oxidative damage may result in further oxidative stress, contributing to neuronal dysfunction, and ultimately, neurodegeneration (Vazquez-Villasenor et al., 2021). When mtDNA is damaged, cells initiate a series of complex repair mechanisms to address the issue. Among these mechanisms, PARP1 plays a crucial role. PARP1 is an enzyme located in both the nucleus and mitochondria, capable of sensing DNA damage and responding rapidly. Following the occurrence of DNA damage, PARP1 is swiftly activated and commences its essential enzymatic activity—catalyzing PARylation (Han et al., 2019). This process involves the transfer of ADP ribose units to proteins, resulting in the formation of poly(ADP-ribose) (PAR) chains. These chains not only facilitate the repair of damaged DNA but also function as signaling molecules that recruit additional repair proteins to the site of damage, thereby initiating a complex network of DNA damage repair (Smith et al., 2019). However, IAA activates cleaved PARP1 (89 kDa), which promotes nerve cell death rather than repair (Maher and Hanneken, 2008). The 116 kDa form refers to the full-length variant of PARP1, representing the original protein’s molecular weight without any cleavage or modifications (Ping et al., 2024). Full-length PARP1 (116 kDa) possesses complete catalytic activity in cells and is involved in various biological processes, including DNA repair and transcriptional regulation. Thus, IAA promotes mtDNA damage rather than DNA repair.

### 2.2 Cellular level

#### 2.2.1 IAA induced decreased mitochondrial membrane potential of nerve cells

IAA inhibits GAPDH and reduces ATP to perturb Na^+^, K^+^ ions in cells (Gemba et al., 1994). Furthermore, IAA induced a decrease in mitochondrial membrane potential of nerve cells (Zhou et al., 2015; Ondricek et al., 2012). The reduction in the potential of the mitochondrial membrane due to disturbances in energy metabolism can significantly impact cellular functions. This decline in mitochondrial membrane potential shows a strong correlation with neurodegenerative disorders. It is essential to keep cellular energy metabolism balance to ensure the stability of mitochondrial membrane potential and preserve the proper functioning of neural cells. A decline in mitochondrial membrane potential results in dysfunction of the electron transport chain located in the mitochondria, which heightens the generation of free radicals and reactive oxygen species ROS. The potential difference across the cell membrane, known as the cell membrane potential, is primarily upheld by ion pumps and channels located within the membrane. This potential is essential for processes such as nerve conduction and intercellular signaling. An imbalance in energy metabolism may lead to decreased ATP production, subsequently affecting the function of sodium-potassium pumps embedded in the cell membrane. These pumps play a crucial role in regulating the concentrations of sodium and potassium ions both inside and outside the cell, and rely on ATP for the energy necessary to operate. When there is a lack of ATP, the functionality of the sodium-potassium pump diminishes, causing sodium ions to accumulate within the cell while extracellular potassium levels drop, ultimately resulting in a decline in the cell membrane potential (Bartolome et al., 2013; Zugno et al., 2003). The decrease in membrane potential further leads to a decrease in ATP production (Wang et al., 2003; Elmi et al., 2001). IAA inhibits GAPDH and reduces ATP to perturb Na^+^, K^+^ ions in cells (Gemba et al., 1994). This is the main reason for the disturbance of cell membrane potential caused by IAA.

#### 2.2.2 IAA causes neuroinflammation

Neuroinflammation is closely linked to the neurotransmitter system and plays a crucial role in the onset and progression of neurodegenerative disorders (Davis et al., 2023). Following treatment with IAA, there was an upregulation in the expression levels of Purinergic Receptor P2X4 (P2X4R) (Yang et al., 2021). Recent research has indicated that the heightened neurotoxicity linked to the overexpression of P2X4R plays a significant role in the pathological mechanisms of neurodegenerative conditions, including injury, inflammation, Alzheimer’s disease, Parkinson’s disease, and multiple sclerosis (Ma et al., 2020). IAA also affects the integrity of the BBB through the P2X4R/p38 signaling pathway. Furthermore, the expression of IκBα was found to be reduced in neurons that were treated with IAA (Kratsovnik et al., 2005). The degraded IκBα activates both subunits of NF-κB, transitioning them from an inactive state to an active one. This process facilitates their movement from the cytoplasm into the nucleus, where they attach to specific genes associated with inflammation, leading to the initiation of transcription for inflammatory cytokines and the induction of inflammation.

#### 2.2.3 IAA causes BBB disruption

**FIGURE 2:**
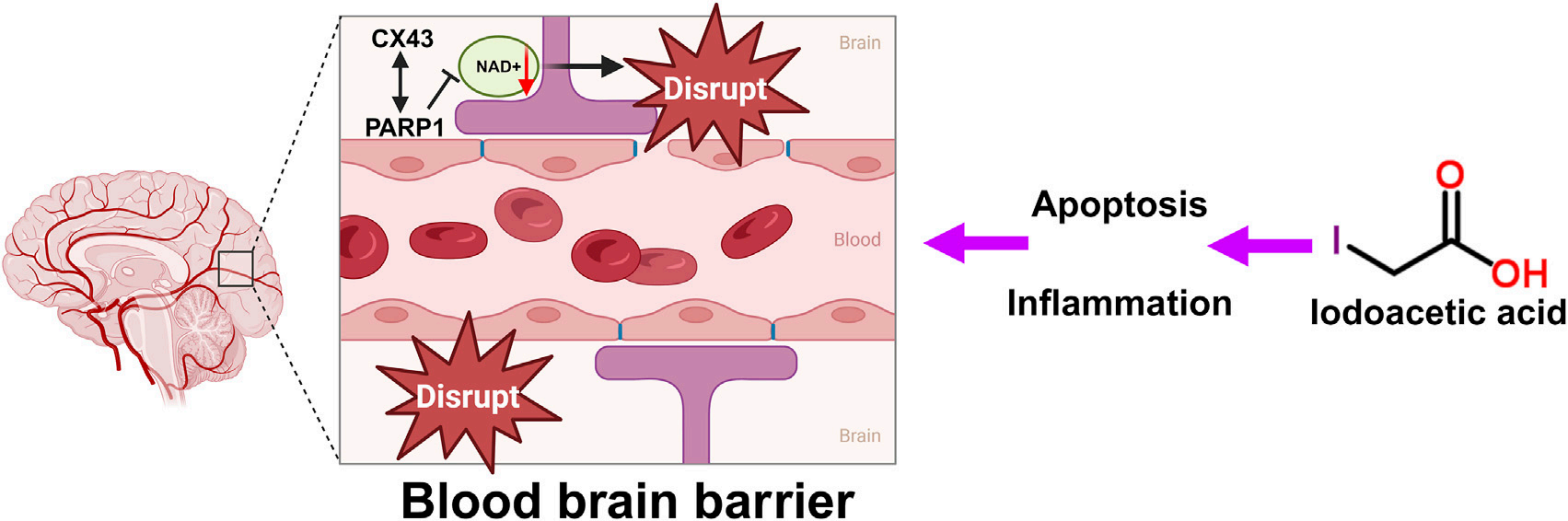
Schematic illustration of the BBB disruption, oxidative stress and DNA damage by IAA. DNA damage caused by IAA-mediated inflammation and oxidative stress disrupts the tight junctions and gap junctions of the BBB. CX43 directly interacts with PARP1. Excessive consumption of NAD+, which affects the integrity of the BBB.

The BBB serves as a protective shield that stops harmful and toxic substances from accessing and damaging brain tissue, thereby fulfilling a crucial function (Wang et al., 2024). Harm to the BBB allows more harmful substances and immune cells to infiltrate the brain, which further worsens nerve cell injury. Following treatment with IAA, the levels of the tight junction-associated protein Connexin43 (Cx43) were reduced (Kim et al., 2023). Cx43 is crucial in the central nervous system (CNS). It is the most prevalent connexin found in the CNS, primarily located in astrocytes, and it exists as gap junctions and hemichannels, facilitating both intercellular and intracellular communication (Zhan et al., 2023). CX43 is a crucial protein involved in the BBB (Zhang et al., 2023). The BBB is formed by brain capillary endothelial cells, pericytes, and the pseudopodia of glial cells, functioning to allow the passage of nutrients while preventing harmful substances in the blood from entering the brain (Wang et al., 2024). CX43 plays a significant role in maintaining the integrity and function of the BBB (Chen et al., 2017b). Studies have shown that with aging, the expression level of CX43 in BBB-related cell subpopulations decreases significantly (Wilson et al., 2008), indicating a close relationship between CX43 expression and the integrity of the BBB (Zhan et al., 2023). Furthermore, CX43 directly interacts with PARP1, a NAD + -consuming enzyme. Enhanced activity of PARP1 leads to excessive consumption of NAD+, which may adversely affect the integrity of the BBB (Zhan et al., 2023). The specific mechanism of IAA caused BBB disruption is shown in Figure 2.

#### 2.2.4 IAA induced neuronal apoptosis

A reduction in the potential of the mitochondrial membrane serves as a critical indicator of apoptosis. As the mitochondrial membrane potential drops, this triggers the opening of the mitochondrial permeability transition pore, resulting in the release of cytochrome C and various other apoptotic factors from the mitochondria into the cytoplasm. This event activates the subsequent apoptotic signaling pathways, ultimately causing cell apoptosis (Rehfeldt et al., 2021). Reduced ATP levels, diminished membrane potential, oxidative stress, heightened DNA damage, and lowered neurotransmitter levels in nerve cells treated with IAA contribute to apoptosis. The molecular mechanisms behind IAA-induced neuronal apoptosis involve the upregulation of mitogen-activated Protein Kinase (MAPK) and JNK, where pMAPK can enhance the expression of TNF, consequently triggering cell apoptosis (Lapchak et al., 2011; Rehfeldt et al., 2021). Furthermore, following the treatment of RGC-5 cells with IAA, there was an elevation in the levels of cysteinyl aspartate specific proteinase-3 (Caspase-3) and Caspase-7 proteins (Ondricek et al., 2012; Gatson and Singh, 2007). Caspase-3 play crucial roles in the apoptosis pathway, working alongside Caspase-7 (Eslami et al., 2020) to facilitate this process. Additionally, treatment with IAA led to an upregulation of cleaved PARP1 expression in RGC-5 cells (Maher and Hanneken, 2008). The generation of cleaved PARP1 is also driven by Caspase-3, which cleaves PARP, leading to its activation, and subsequently triggers other proteases associated with apoptosis to conclude the apoptotic process (Ping et al., 2024). A decrease in the Bcl-2/Bax ratio was observed in rat pheochromocytoma cells (PC12), that were treated with IAA (Chen et al., 2017a). Bcl-2 and Bax modulate the opening of channels induced by the apoptotic activator Bax dimer on the mitochondrial membrane, enhancing permeability by managing the mitochondrial membrane’s permeability. A reduction in the Bcl-2/Bax ratio signifies an increase in apoptosis.

**FIGURE 3:**
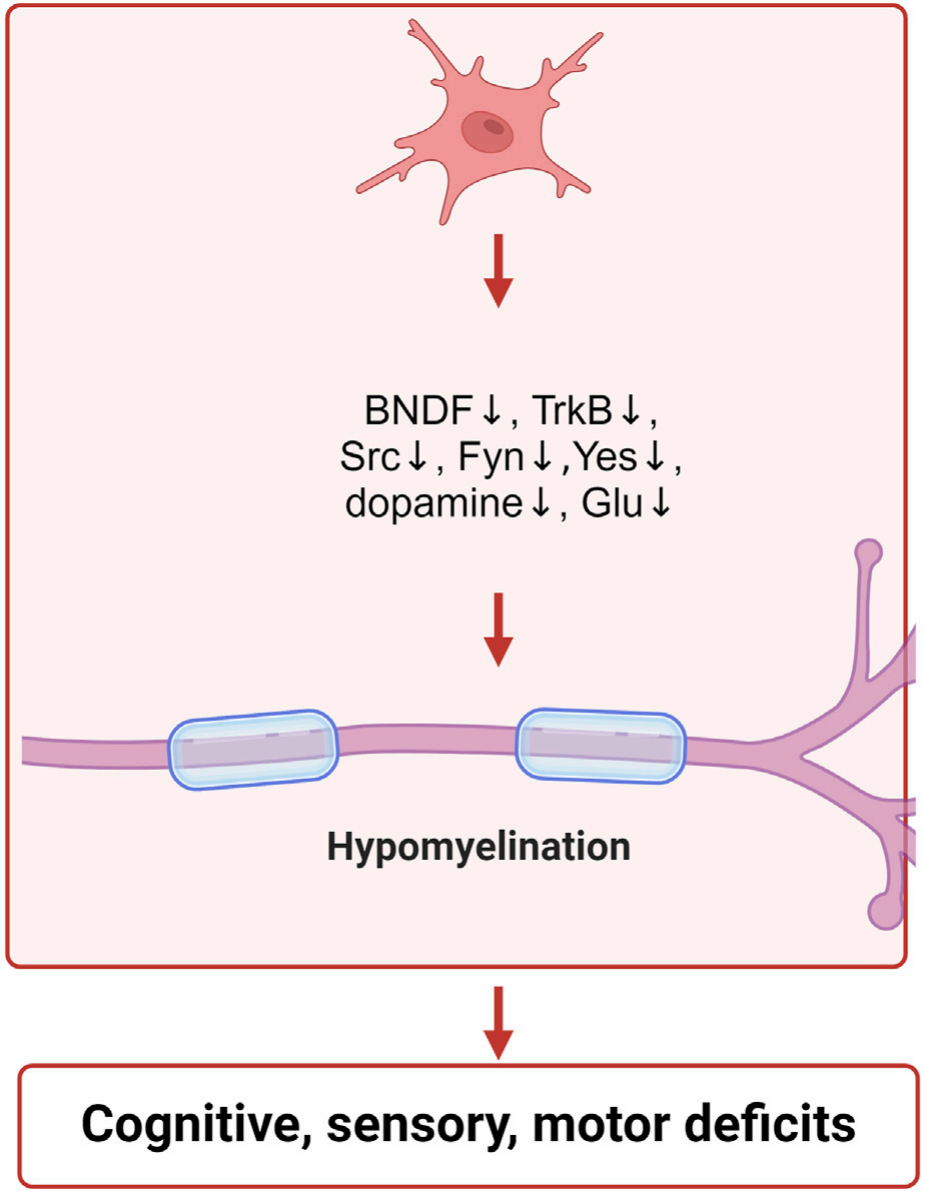
Schematic illustration of the neurotoxic mechanisms of IAA inclued neurotransmitter disorders and neurodevelopment. IAA inhibits the expression of BNDF, TrkB, Src, Fyn, Yes, and reduces the levels of dopamine and glutamate, leading to neurodevelopmental abnormalities.

Furthermore, following IAA treatment, the levels of p-AKT, p-mTOR, p-p70, and p-GSK3β were found to be decreased (Cho et al., 2021; Mata and Goldberg, 2023). Akt plays a crucial role in the growth of cells. It serves as a key modulator of cell survival by directly suppressing proteins that promote apoptosis (Hiromura et al., 2004). p-mTOR is an essential molecule that regulates growth by detecting and interacting with various nutritional and environmental elements, such as growth factors, energy availability, cellular stress, and amino acids. It integrates these signals to stimulate cell growth through the phosphorylation of substrates, thereby promoting anabolism or suppressing catabolism (Yao et al., 2016). p70 is essential in regulating the cell cycle, growth, and survival. One of the primary mechanisms for managing cell survival, proliferation, and metabolism is the PI3K/mTOR signaling pathway (Gao et al., 2003). GSK-3β has the ability to modulate Bax, which subsequently influences mitochondrial permeability and the release of cytochrome C, playing a role in the regulation of apoptosis (Xu et al., 2023). The decline in mitochondrial membrane potential over an extended period, alongside energy shortages and cellular apoptosis, is associated with the development of various neurodegenerative disorders, including Alzheimer’s and Parkinson’s diseases. In these conditions, both mitochondrial dysfunction and apoptosis represent widespread pathological characteristics (Rehfeldt et al., 2021). The specific mechanism of IAA neurotoxicity is shown in Figure 3.

### 2.3 Neurological manifestations

#### 2.3.1 IAA causes neurotransmitter disorders

Neurons depend on the energy supplied by mitochondria to sustain regular neurotransmitter release. A reduction in mitochondrial membrane potential results in an inadequate energy supply, impacting the production and release of neurotransmitters, and consequently influencing the transmission of nerve signals (Sun et al., 2016). IAA inhibits dopamine levels in cerebellar Purkinje cells (LoPachin et al., 2006). One of the key pathological alterations observed in Parkinson’s disease is the loss of dopaminergic neurons located in the substantia nigra of the midbrain. This neuronal death results in a reduction of dopamine levels within the striatum, which serves as the brain’s center for motor control, ultimately causing impairment in the striatal regulation of motor functions (Salamon et al., 2020). Moreover, ATP serves not only as an energy source for tissue cells but also acts as a neurotransmitter that has excitatory properties (Edwards and Gibb, 1993). Glutamic acid (Glu) is recognized broadly as a molecule involved in metabolism, yet it also functions as a neurotransmitter (Franco et al., 2021). IAA enters the brain to inhibit glycolysis and reduce the levels of ATP and Glu in nerve cells (Sperling et al., 2003; Suna et al., 2009; Maher and Hanneken, 2008; Sabri, 1994; Rogel et al., 2005; Gemba et al., 1994). ATP synthase in mitochondria plays a crucial role in generating cellular ATP; ATP synthase accomplishes this by harnessing the mitochondrial membrane potential created through the ongoing oxidation of particular cellular metabolites (Ebanks and Chakrabarti, 2022). The process of releasing, binding, reabsorbing, and breaking down glutamate in the brain plays a significant role in both physiology and pathophysiology. Notably, alterations in glutamatergic neurotransmission become more pronounced during neurodegenerative disorders, correlating with cognitive deficits that manifest as challenges in memory, learning, attention, and decision-making (Castillo-Vazquez et al., 2024). Intracellular molecules known as neurotransmitters are essential for amplifying, transmitting, and converting signals, significantly impacting information transfer within the nervous system. These compounds influence various functions such as mood, cognition, memory, learning, and motor activity. Consequently, an imbalance in the homeostasis of neurotransmitters is increasingly linked to numerous neurological and neurodegenerative disorders (Teleanu et al., 2022).

#### 2.3.2 IAA inhibits neurodevelopment

GAPDH is also crucial for the growth and differentiation of neurons. In mice, the inhibition of the GluA2-GAPDH interaction adversely affects neurodevelopment. The GluA2 subunit is essential for the development of cortical neurons due to its association with GAPDH (Lee et al., 2018). The expressions of brain-derived neurotrophic factor (BDNF), tyrosine receptor kinase B (TrkB), SRC proto-oncogene (Src), FYN proto-oncogene (Fyn) and YES proto-oncogene 1 (Yes) were decreased after IAA treatment (Cho et al., 2021; Shani et al., 2009; Gatson and Singh, 2007). BDNF aids in the maintenance of neurons and brain cells, fosters synaptic connections among neurons, and plays a crucial role in learning as well as the storage of long-term memories (Klein et al., 2011). The receptor for BDNF is TrkB, which is an essential component of the BDNF-TrkB signaling pathway. BDNF performs a distinctive biological role by interacting with its receptor, TrkB, which primarily contributes to neuronal development and is crucial for signal transduction. This pathway encompasses the swift modulation of excitatory and inhibitory synaptic transmission in neurons located in the hippocampus (Otani et al., 2017; Rantamäki et al., 2011). The SRC, YES, and FYN genes produce three related tyrosine protein kinases that are found in human neural tissues (Pyper and Bolen, 1989). The roles of SRC, YES, and FYN are significant in synaptic transmission and the processes underlying excitatory synaptic plasticity (Kalia and Salter, 2003). Src family kinases are involved in the regulation of signaling pathways that contribute to the formation of neuromuscular synapses (Stein et al., 1994). The non-receptor Src tyrosine kinase is a crucial element in intracellular signal transduction and is highly expressed within the nervous system (Zhao et al., 2003). Src has the ability to enhance the proliferation and dedifferentiation of Schwann cells, aiding in the repair of peripheral nerve injuries (Govindasamy et al., 2022). Fyn, a cytoplasmic tyrosine kinase, is essential for various biological processes. Within both the central and peripheral nervous systems, Fyn is pivotal for initiating myelination through the formation of myelinated glial cells, such as Schwann cells and oligodendrocytes (Torii et al., 2017). Furthermore, Fyn has been shown to facilitate the regulation of the growth of axons and dendrites (Liu et al., 2006). The Fyn protein, associated with myelin, plays a crucial role in starting the myelination process and is significant in the overall myelination mechanism (Biffiger et al., 2000). IAA treatment led to the inhibition of nerve cell growth and differentiation, which was closely associated with the suppression of neurodevelopmental proteins by IAA. The specific mechanism of IAA neurotoxicity is shown in Figure 3.

## 3 Discussion and conclusion

**FIGURE 4:**
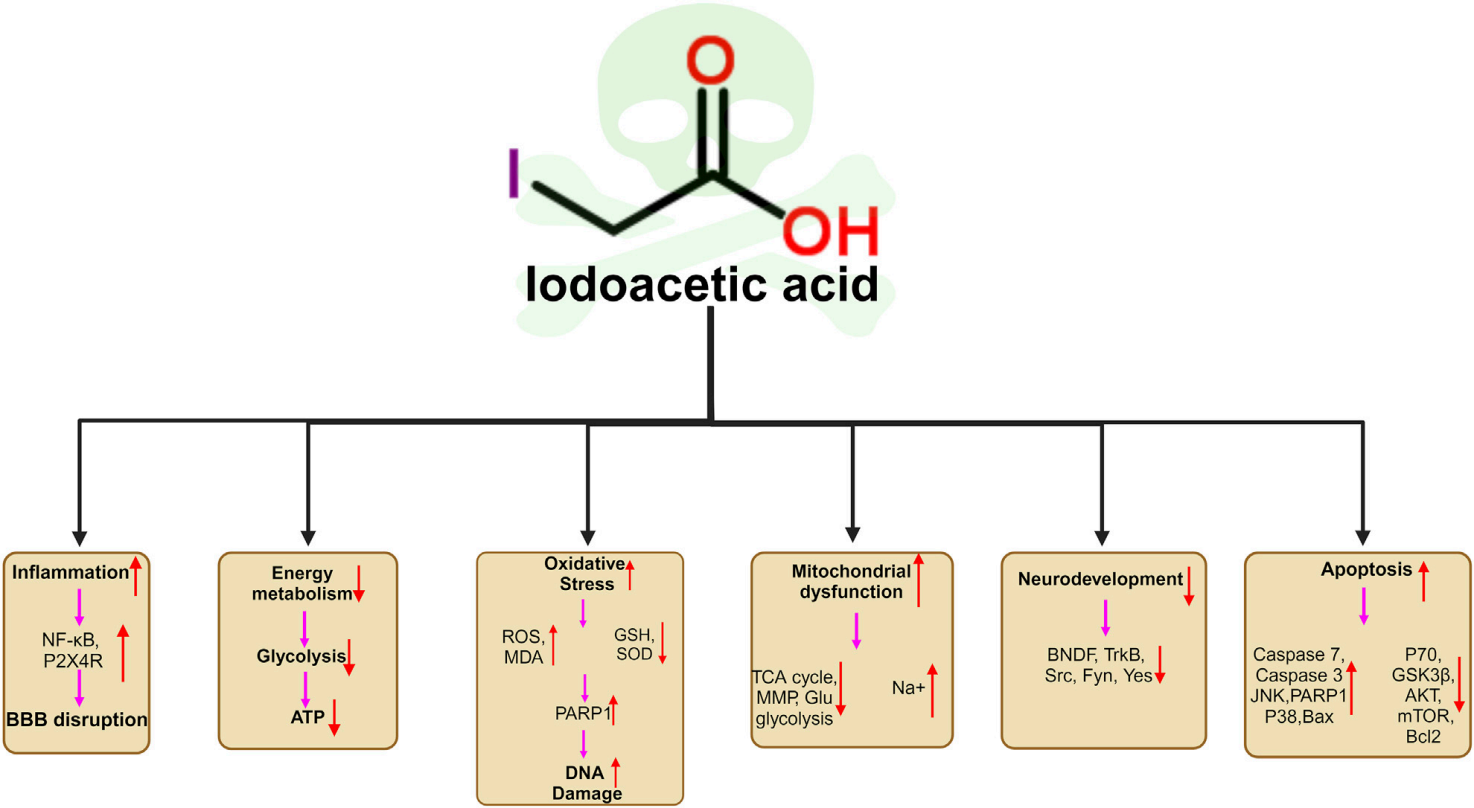
Schematic illustration of the neurotoxic mechanisms of IAA. (1) IAA promoted neuroinflammation by promoting the expression of NFκB, and P2X4R. (2) IAA caused Energy metabolism disorder. (3) The neurotoxicity of IAA caused oxidative stress and DNA damage by stimulating the increase of ROS and reducing the level of antioxidant SOD, GSH (4) IAA induced mitochondrial dysfunction and ATP production. (5) IAA neurotoxicity cause nerve cell developmental disorders, and abnormal differentiation by reducing the BNDF, TrkB,Src, Fyn, Yes expression. (6) IAA cause nerve cell apoptosis by reducing anti-apoptotic proteins P70, GSK3B, AKT, mTOR, Bcl2 expression and promoting apoptosis protein JNK, PARP1, Caspase3, Caspase7, Bax, MAPK, PARP1, Bax expression.

As most studies of IAA have focused on the fields of genotoxicity, carcinogenicity and reproductive toxicity, few studies have been conducted on neurotoxicity. And there has been no review of IAA neurotoxicity. For the first time, we review the neurotoxicity of IAA. IAA causes neurotoxicity through a variety of mechanisms, including oxidative stress, DNA damage, inflammation, BBB disruption, and apoptosis. Neurotoxic manifestations include neurodevelopmental disorders (abnormal neural differentiation and demyelination), neurotransmitter disorders, etc. The unavoidable limitation of this review is that most of the studies are based on cell experiments and there are few studies on neurotoxicity in mammals. In addition, there are no studies that have confirmed neurotoxicity caused by daily intake of IAA through drinking water. This may be related to the daily dose of IAA ingested. However, oxidative stress and inflammation in nerve cells caused by long-term low level of IAA intake are closely related to neurodegeneration. Future experimental studies should consider these factors. Our review provides insights into the mechanism of IAA neurotoxicity. The molecular mechanism of neurotoxicity induced by IAA is shown in Figure 4.

## Abbreviations

adenosine Triphosphate, ATP; brain-derived neurotrophic factor, BDNF; central nervous system, CNS; Connexin43, Cx43; cysteinyl aspartate specific proteinase-3, Caspase-3; disinfection by-products, DBPs; FYN proto-oncogene, Fyn; Glutamic acid, Glu; glutathione, GSH; glyceraldehyde-3-phosphate dehydrogenase, GAPDH; Iodoacetic acid, IAA; lactate dehydrogenase, LDH; mitogen-activated Protein Kinase, MAPK; mitochondrial membrane potential, MMP; pheochromocytoma cells, PC12; Poly(ADP-ribose) polymerase 1, PARP1; Purinergic Receptor P2X4, P2X4R; reactive nitrogen species, RNS; reactive oxygen species, ROS; retinal ganglion cells, RGC-5 cells; RSK, ribosomal s6 kinase; SRC proto-oncogene, Src; superoxide dismutase, SOD; tricarboxylic acid, TCA; tyrosine receptor kinase B, TrkB; FYN proto-oncogene: Fyn.
